# Cardio-miRNAs and onco-miRNAs: circulating miRNA-based diagnostics for non-cancerous and cancerous diseases

**DOI:** 10.3389/fcell.2014.00061

**Published:** 2014-10-16

**Authors:** Masaru Katoh

**Affiliations:** Department of Omics Network, National Cancer CenterTokyo, Japan

**Keywords:** Alzheimer's disease, early diagnosis, gastric cancer, hypertension, pancreatic cancer, personalize medicine, rheumatoid arthritis, stem cells

## Abstract

Cardiovascular diseases and cancers are the leading causes of morbidity and mortality in the world. MicroRNAs (miRNAs) are short non-coding RNAs that primarily repress target mRNAs. Here, miR-24, miR-125b, miR-195, and miR-214 were selected as representative cardio-miRs that are upregulated in human heart failure. To bridge the gap between miRNA studies in cardiology and oncology, the targets and functions of these miRNAs in cardiovascular diseases and cancers will be reviewed. ACVR1B, BCL2, BIM, eNOS, FGFR3, JPH2, MEN1, MYC, p16, and ST7L are miR-24 targets that have been experimentally validated in human cells. ARID3B, BAK1, BCL2, BMPR1B, ERBB2, FGFR2, IL6R, MUC1, SITR7, Smoothened, STAT3, TET2, and TP53 are representative miR-125b targets. ACVR2A, BCL2, CCND1, E2F3, GLUT3, MYB, RAF1, VEGF, WEE1, and WNT7A are representative miR-195 targets. BCL2L2, ß-catenin, BIM, CADM1, EZH2, FGFR1, NRAS, PTEN, TP53, and TWIST1 are representative miR-214 targets. miR-125b is a good cardio-miR that protects cardiomyocytes; miR-195 is a bad cardio-miR that elicits cardiomyopathy and heart failure; miR-24 and miR-214 are bi-functional cardio-miRs. By contrast, miR-24, miR-125b, miR-195, and miR-214 function as oncogenic or tumor suppressor miRNAs in a cancer (sub)type-dependent manner. Circulating miR-24 is elevated in diabetes, breast cancer and lung cancer. Circulating miR-195 is elevated in acute myocardial infarction, breast cancer, prostate cancer and colorectal adenoma. Circulating miR-125b and miR-214 are elevated in some cancers. Cardio-miRs and onco-miRs bear some similarities in functions and circulation profiles. miRNAs regulate WNT, FGF, Hedgehog and other signaling cascades that are involved in orchestration of embryogenesis and homeostasis as well as pathogenesis of human diseases. Because circulating miRNA profiles are modulated by genetic and environmental factors and are dysregulated by genetic and epigenetic alterations in somatic cells, circulating miRNA association studies (CMASs) within several thousands of cases each for common non-cancerous diseases and major cancers are necessary for miRNA-based diagnostics.

## Introduction

MicroRNAs (miRNAs) are short non-coding RNAs that primarily repress protein expression from target mRNAs with imperfect or perfect complementarity through mRNA degradation and translational inhibition or mRNA cleavage, respectively (Kasinski and Slack, 2011; van Rooij and Olson, 2012). For example, miR-15, miR-16, miR-20a, and miR-20b are anti-angiogenic miRNAs that repress VEGFA (VEGF) (Wang and Olson, 2009; Katoh, 2013b). miR-200 family members inhibit epithelial-to-mesenchymal transition (EMT) and self-renewal of stem cells through repression of ZEB1/2 and BMI1, respectively (Katoh and Katoh, 2008; Oishi et al., 2012; Feng et al., 2014). miRNAs regulate a variety of cellular processes, such as stemness, proliferation, senescence, apoptosis, inflammatory cytokine production, EMT, metastasis and drug resistance.

**Figure 1:**
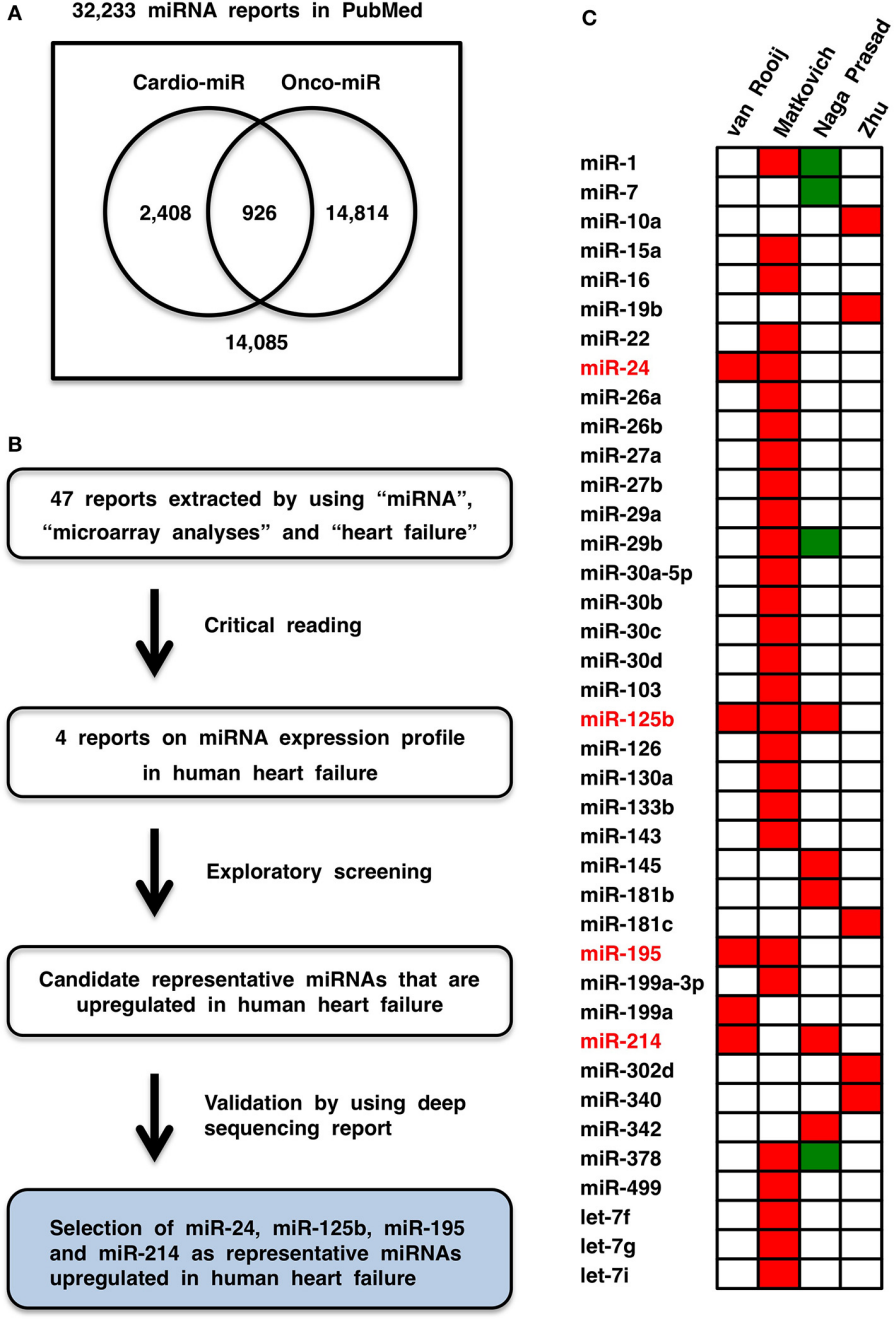
**Cardio-miRNAs upregulated in human heart failure**. **(A)** Bibliology of miRNAs, cardio-miRs, and onco-miRs. **(B)** Flowchart of representative cardio-miR selection. Based on exploratory screening of miRNA expression reports (van Rooij et al., 2006; Matkovich et al., 2009; Naga Prasad et al., 2009; Zhu et al., 2013) and validation process utilizing next-generation sequence report (Leptidis et al., 2013), miR-24, miR-125b, miR-195, and miR-214 were selected as representative cardio-miRs that are upregulated in human heart failure. **(C)** Heat map of miRNA expression profiles in human heart failure. Red, upregulated; green, downregulated.

Cardiovascular diseases and cancers are the leading causes of morbidity and mortality in the world (Lozano et al., 2012). miRNAs involved in heart diseases (Divakaran and Mann, 2008), vascular diseases (Wang and Olson, 2009) and cancers (Croce, 2009) are designated cardio-miRs, angio-miRs, and onco-miRs, respectively, and the same miRNA can function as a cardio-miR, angio-miR or onco-miR in a context-dependent manner. Among 32,233 miRNA manuscripts in the PubMed database, 3334 and 15,740 manuscripts were extracted by using cardiovascular and oncological terms, respectively, and only 926 manuscripts were extracted by using both terms (Figure 1A), which indicates that the outcomes of miRNA studies might not be efficiently shared between the different disciplines. To bridge the gap between miRNA studies in cardiology and oncology, representative cardio-miRs upregulated in human heart failure were selected based on database screening. The targets and functions of these miRNAs in cardiovascular diseases and cancers are comprehensively reviewed, and then circulating miRNA-based diagnostics for non-cancerous and cancerous diseases are discussed with a focus on personal diversity related to genetic and environmental factors.

### Representative cardio-miRs upregulated in heart failure

Heart failure is a progressive decline in cardiac functions that occurs at the end stage of cardiovascular diseases, such as ischemic heart disease, hypertension and diabetes (Hill and Olson, 2008; Shah and Mann, 2011; Zhou et al., 2013b). Myocardial infarction is caused by coronary artery occlusion, which leads to the death of cardiomyocytes in the infarcted region owing to insufficient oxygen supply. Ischemic stress occurs in surviving cardiomyocytes in the surrounding or peripheral area of an infarcted region, and then hypertrophic growth of myocardiocytes and interstitial fibrosis occur in the non-infarcted region of the heart. By contrast, persistent pressure overload causes cardiac wall thickening of the left ventricle and hypertrophic growth of cardiomyocytes. Cardiac hypertrophy leads to maladaptive remodeling of the left ventricle and eventually results in patient death owing to fatal arrhythmia and/or heart failure.

Forty-seven reports were recovered by initial screening of the literature in the PubMed and Web of Science (WoS) databases by using “heart failure,” “miRNA or miRNAs,” and “microarray.” Then, four reports on microarray analyses (van Rooij et al., 2006; Matkovich et al., 2009; Naga Prasad et al., 2009; Zhu et al., 2013) were selected by critical reading (Figure 1B). Based on the criterion “miRNA that is upregulated in at least two reports on microarray analyses,” miR-24, miR-125b, miR-195, and miR-214 were selected as candidate representative cardio-miRs that are upregulated in human heart failure (Figure 1C). Because data obtained by using microarray analyses are not always correct, upregulation of miR-24, miR-125b, miR-195, and miR-214 in human heart failure were then validated by using a deep sequencing report on miRNA profiles in human heart failure (Leptidis et al., 2013). Based on the exploration and validation processes, miR-24, miR-125b, miR-195, and miR-214 were designated the representative cardio-miRs upregulated in human heart failure (Figure 1B).

### miR-24

#### Human chromosomal loci of miR-24 genes

**Figure 2:**
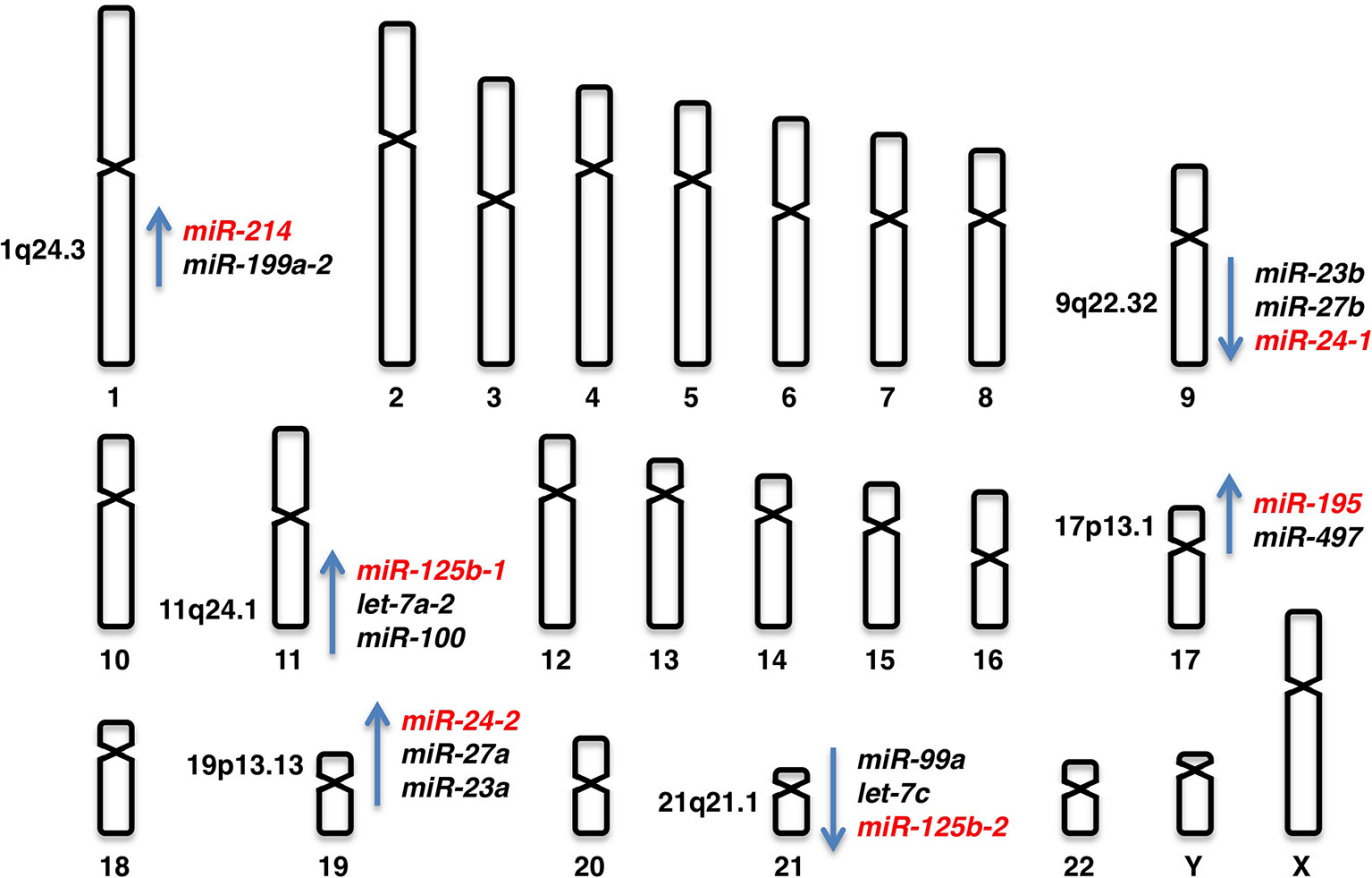
**Human chromosomal loci of *miR-24-1, miR-24-2, miR-125b-1, miR-125b-2, miR-195*, and *miR-214***.

*miR-24* is derived from the *miR-23b/miR-27b/miR24-1* locus at human chromosome 9q22.32 and the *miR-23a/miR-27a/miR-24-2* locus at human chromosome 19p13.13 (Figure 2).

#### Targets of miR-24

miRNA targets demonstrated in rodents are not always conserved in humans owing to species divergence (Le et al., 2009), while putative miRNA targets predicted by using bioinformatics tools, such as TargetScan (http://www.targetscan.org), PicTar (http://pictar.mdc-berlin.de) and miRanda (http://www.microrna.org), are not always true. In this review, miRNA targets validated in human cells are listed up (Table 1).

**Table 1:** **Validated targets of miR-24, miR-125b, miR-195, and miR-214**.

**miR-24**	**miR-125b**	**miR-195**	**miR-214**
ACVR1B	ARID3B	ACVR2A	ASF1B
ARHGAP19	BAK1	ARL2	BCL2L2
AURKB	BCL2	BCL2	β-catenin
BCL2	BCL2L2	BCL2L2	BIM
BIM	BMPR1B	BIRC5	CADM1
CCNA2	CBFB (CBFβ)	CCND1	CCL5
CDC2	CDH5	CCNE1	CD276 (B7-H3)
CDK4	CDKN2A (p14)	CDC42	EZH2
CDKN1B (p27)	DICER1	CDK4	FGFR1
CDKN2A (p16)	E2F3	CDK6	GALNT7
DHFR	EDN1	E2F3	HDGF
DIAPH1	EPO	GLUT3	ING4
DUSP16	EPOR	IKKα	ITGA3
E2F2	ERBB2	MYB	LTF
eNOS (NOS3)	ERBB3	RAF1	LZTS1
FAF1	ETS1	TAB3	MAP2K3
FEN1	FGFR2	VAV2	MAPK8
FGFR3	IL6R	VEGF	NRAS
GATA2	IRF4	WEE1	PSMD10
H2AFX	JUN (c-Jun)	WNT7A	PTEN
HNF4A	LIN28A		TFAP2C
JPH2	LIN28B		TP53
LIMK2	MCL1		TWIST1
MEN1	MMP13		UBE2I
MYC (c-Myc)	MUC1		XBP1
NET1	NCOR2		
PAK4	PGF		
PTPN9	PRDM1		
PTPRF	SIRT7		
RASA1	Smoothened		
SH3PXD2A	ST18		
SLC4A1	STARD13		
SPRY2	STAT3		
ST7L	TET2		
TRIB3	TNF (TNF-α)		
XIAP	TNFSF4		
ZNF217	TP53		

ACVR1B (Activin receptor 1B) (Wang et al., 2008a), ARHGAP19 (Amelio et al., 2012), AURKB (Aurora kinase B) (Lal et al., 2009a), BCL2 (Srivastava et al., 2011), BCL2L11 (pro-apoptotic BIM) (Qian et al., 2011), CCNA2 (Cyclin A2) (Lal et al., 2009a), CDC2 (Lal et al., 2009a), CDK4 (Cyclin-dependent kinase 4) (Lal et al., 2009a), CDKN1B (p27 KIP1) (Giglio et al., 2013), CDKN2A (p16 INK4a) (Lal et al., 2008), DHFR (Dihydrofolate reductase) (Mishra et al., 2007), DIAPH1 (Diaphanos homolog 1) (Zhou et al., 2013a), DUSP16 (MKP7) (Zaidi et al., 2009), E2F2 (Lal et al., 2009a), eNOS (NOS3) (Meloni et al., 2013), FAF1 (Fas-associated factor 1) (Qin et al., 2010), FEN1 (Lal et al., 2009a), FGFR3 (FGF receptor 3) (Rio-Machin et al., 2013), GATA2 (Fiedler et al., 2011), H2AFX (Histone H2AX) (Lal et al., 2009b), HNF4A (HNF4α) (Takagi et al., 2010), JPH2 (Junctophilin 2) (Xu et al., 2012b), LIMK2 (LIM-domain kinase 2) (Zhou et al., 2013a), MEN1 (Luzi et al., 2012), MYC (c-Myc) (Lal et al., 2009a), NET1 (NET1A or ARHGEF8) (Papadimitriou et al., 2012), PAK4 (Fiedler et al., 2011), PTPN9 (Protein tyrosine phosphatase, non-receptor type 9) (Du et al., 2013), PTPRF (Protein tyrosine phosphatase, receptor type F) (Du et al., 2013), RASA1 (Ras GAP) (Fiedler et al., 2011), SH3PXD2A (TSK5) (Amelio et al., 2012), SLC4A1 (Anion exchanger 1) (Wu et al., 2010), SPRY2 (Sprouty homolog 2) (Li et al., 2013a), ST7L (Chen et al., 2013a), TRIB3 (Tribbles pseudokinase 3) (Chan et al., 2010), XIAP (X-linked inhibitor of apoptosis) (Xie et al., 2013), and ZNF217 (Zinc finger protein 217) (Szczyrba et al., 2013) are all validated targets of miR-24 (Table 1).

#### Involvement of miR-24 in cardiovascular diseases

**Figure 3:**
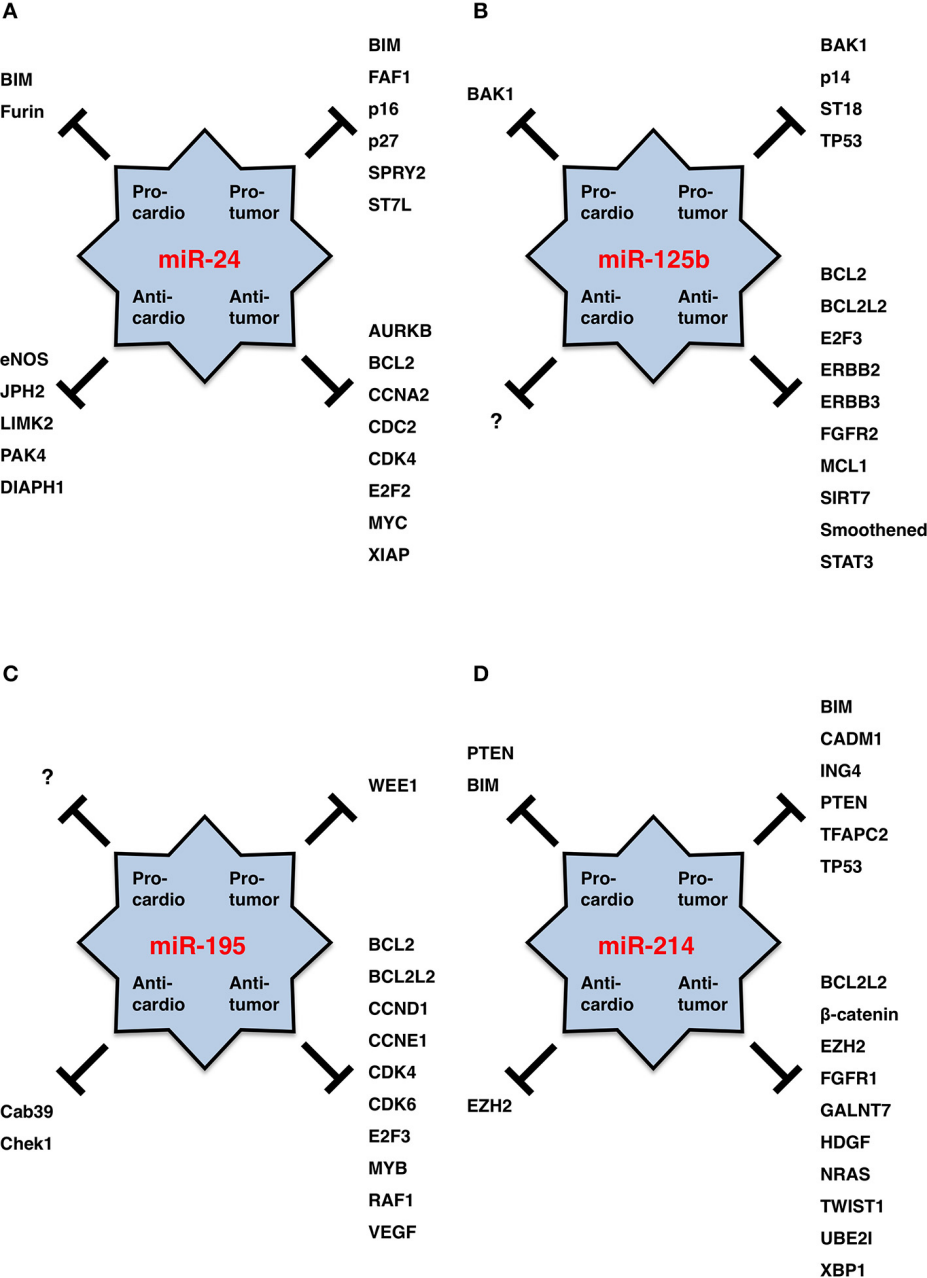
**miRNA functions in cardiology and oncology**. **(A)** miR-24. **(B)** miR-125b. **(C)** miR-195. **(D)** miR-214. Targets for pro-tumor, anti-tumor, pro-cardio and anti-cardio functions of each miRNA are shown. miR-125b is a good cardio-miR that protects cardiomyocytes. miR-195 is a bad cardio-miR that elicits cardiomyopathy and heart failure. miR-24 and miR-214 are bi-functional cardio-miRs. By contrast, miR-24, miR-125b, miR-195, and miR-214 function as oncogenic or tumor suppressor miRNAs in a cancer (sub)type-dependent manner.

miR-24 is upregulated in ischemic heart endothelial cells as a result of hypoxia-induced HIF-dependent transcription, but it is then transiently downregulated in adjacent surviving regions of acute myocardial infarction owing to the recovery of blood supply (Fiedler et al., 2011; Qian et al., 2011; Camps et al., 2014). miR-24 promotes cardiomyocyte survival through repression of pro-apoptotic Bim (Qian et al., 2011) and reduces cardiac fibrosis through repression of Furin protease that controls the activation of latent TGFβ (Wang et al., 2012b). On the other hand, miR-24 inhibits the survival, migration, proliferation and tube formation of endothelial cells (angiogenesis) through repression of eNOS and actin cytoskeleton regulators, such as DIAPH1, LIMK2, and PAK4 (Fiedler et al., 2011; Meloni et al., 2013; Zhou et al., 2013a). miR-24 is upregulated in the chronic phase after myocardial infarction and promotes hypertrophic growth of cardiomyocytes in mouse model experiments and disturbs cardiac contraction through repression of JPH2 that is involved in the excitation-contraction coupling process of the heart (van Rooij et al., 2006; Xu et al., 2012b). Because miR-24 protects cardiomyocytes themselves and reduces cardiac fibrosis but inhibits angiogenesis and deteriorates heart failure, miR-24 is a multi-functional cardio-miR that plays good and bad roles in heart failure (Figure 3A).

#### Involvement of miR-24 in cancers

miR-24 is transcriptionally upregulated in acute myeloid leukemia (AML) with t(8;21) by the RUNX1-RUNX1T1 (AML1-ETO) fusion protein, which promotes proliferation and blocks differentiation of myeloid cells through repression of DUSP16 and subsequent activation of mitogen-activated protein kinase (MAKP) signaling (Zaidi et al., 2009). miR-24 is transcriptionally upregulated in breast cancer with lymph node metastasis in part by MYC (Li et al., 2013a), and overexpression of miR-24 in MCF-7 breast cancer cells promotes invasion and metastasis through repression of SPRY2 and subsequent MAPK activation (Li et al., 2013a). miR-24 is upregulated by the E6 and E7 oncoproteins of human papilloma virus type 16 (HPV16), which promotes proliferation through p27 repression (McKenna et al., 2014). Upregulation of miR-24 in glioblastoma promotes survival, proliferation and invasion through repression of tumor suppressor ST7L (Chen et al., 2013a). Upregulation of miR-24 in pancreatic endocrine tumors (Volinia et al., 2006) and parathyroid tumors (Luzi et al., 2012) can contribute to the progression of multiple endocrine neoplasia type 1 (MEN1) syndrome through repression of its causative gene product. miR-24 is also upregulated in colon cancer (Volinia et al., 2006), lung adenocarcinoma (Yanaihara et al., 2006), pancreatic ductal adenocarcinoma (Jamieson et al., 2012) and gastric cancer (Volinia et al., 2006; Bandres et al., 2009). Because pro-tumor miR-24 promotes survival, proliferation and invasion through repression of BIM, FAF1, p16, p27, SPRY2, and ST7L (Figure 3A), oncogenic miR-24 is upregulated in human cancers.

By contrast, miR-24 is downregulated in A549 and H1437 non-small-cell lung cancer cells owing to copy number loss of the *miR-24-2* locus (Xie et al., 2013) (Table 2). miR-24 is also downregulated in prostate cancer (Volinia et al., 2006) and hepatocellular carcinoma (HCC) recurring after liver transplantation (Han et al., 2012). Because anti-tumor miR-24 promotes differentiation, growth arrest and apoptosis through repression of AURKB, BCL2, CCNA2, CDC2, CDK4, E2F2, MYC, and XIAP (Figure 3A), tumor suppressor miR-24 is downregulated in human cancers.

**Table 2:** **Genetic and epigenetic alterations of *miR-24, miR-125b, miR-195*, and *miR-214***.

**miRNA gene**	**Chromosome locus**	**Disease**	**Genetic alteration**	**Epigenetic alteration**	**miRNA expression**
*miR-24-1*	9q22.32				
*miR-24-2*	19p13.13	Lung cancer	Deletion		Down
*miR-125b-1*	11q24.1	AML and MDS	t(2;11)(p21;q24)		Up
		BCP-ALL	t(11;14)(q24;q32)		Up
		Cervical cancer	Deletion		Down
		Breast cancer		Epigenetic silencing	Down
*miR-125b-2*	21q21.1	DS-AMKL	21 trisomy		Up
*miR-195*	17p13.1	Colorectal adenoma	Deletion	Epigenetic silencing	Down
		Colorectal cancer	Deletion		Down
		Breast cancer		Epigenetic silencing	Down
		Gastric cancer		Epigenetic silencing	Down
*miR-214*	1q24.3	Liposarcoma	Gene amplification		Up
		Breast cancer	Deletion		Down

*AML, acute myeloid leukemia; MDS, myelodysplastic syndrome; BCP-ALL, B-cell precursor acute lymphoblastic leukemia; DS-AMKL, Down syndrome with acute megakaryocytic leukemia*.

miR-24 functions as an oncogenic or tumor suppressor miRNA in a cancer (sub)type- or cell line-dependent manner (Figure 3A).

### miR-125b

#### Human chromosomal loci of miR-125b genes

*miR-125b* is derived from the *miR-125b-1* and *miR-125b-2* loci. *miR-125b-1* is clustered with *let-7a-2* and *miR-100* at human chromosome 11q24.1 and *miR-125b-2* is clustered with *let-7c* and *miR-99a* at human chromosome 21q21.1 (Figure 2).

#### Targets of miR-125b

ARID3B (Akhavantabasi et al., 2012), BAK1 (BCL2-antagonist/killer 1) (Shi et al., 2007), BCL2 (Zhao et al., 2012a), BCL2L2 (anti-apoptotic BCL-W) (Gong et al., 2013), BMPR1B (Sætrom et al., 2009), CBFB (Core binding factor β) (Lin et al., 2011), CDH5 (VE-cadherin) (Muramatsu et al., 2013), CDKN2A (p14 ARF) (Amir et al., 2013), DICER1 (Klusmann et al., 2010), E2F3 (Huang et al., 2011a), EDN1 (Endothelin 1) (Li et al., 2010), EPO (Ferracin et al., 2013), EPOR (Ferracin et al., 2013), ERBB2 (Scott et al., 2007), ERBB3 (Scott et al., 2007), ETS1 (Zhang et al., 2011), FGFR2 (Xu et al., 2011), IL6R (Gong et al., 2013), IRF4 (Malumbres et al., 2009), JUN (c-Jun) (Kappelmann et al., 2013), LIN28A (Lin-28) (Wu and Belasco, 2005), LIN28B (Liang et al., 2010), MCL1 (Gong et al., 2013), MMP13 (Xu et al., 2012c), MUC1 (Rajabi et al., 2010), NCOR2 (Yang et al., 2012a), PGF (Placental growth factor) (Alpini et al., 2011), PRDM1 (BLIMP1) (Malumbres et al., 2009), SIRT7 (Kim et al., 2013a), Smoothened (SMO) (Ferretti et al., 2008), ST18 (Klusmann et al., 2010), STARD13 (Tang et al., 2012), STAT3 (Liu et al., 2011), TET2 (Cheng et al., 2013), TNF (TNF-α) (Rajaram et al., 2011), TNFSF4 (Smirnov and Cheung, 2008), and TP53 (Le et al., 2009) are representative targets of miR-25b (Table 1).

#### Involvement of miR-125b in cardiovascular diseases

miR-125b and LIN28A are human homologs of *Caenorhabditis elegans* (*C. elegans*) lin-4 and lin-28, respectively. *C. elegans* lin-4 is involved in the repression of lin-28 to orchestrate morphogenesis during larval stage, whereas human miR-125b is involved in the repression of LIN28A during the differentiation of embryonic stem cells (ESCs) into myocardial precursors and cardiomyocytes (Wu and Belasco, 2005; Wong et al., 2012).

miR-125b is physiologically expressed in perivascular stromal cells rather than cardiomyocytes of the developing mouse heart (Schneider et al., 2011) and in cardiac valves rather than myocardium of the adult rat heart (Vacchi-Suzzi et al., 2013). miR125b is upregulated in mouse cardiac endothelial cells during endothelial-to-mesenchymal transition (EndMT) induced by TGF-ß (Ghosh et al., 2012). In addition, mir-125b is upregulated in early-stage cardiac hypertrophy after aortic banding (Busk and Cirera, 2010) and also in late-stage cardiac hypertrophy and heart failure (van Rooij et al., 2006). Ectopic miR-125b expression by using adenovirus vector does not elicit cardiomyocyte hypertrophy *in vitro* (van Rooij et al., 2006), whereas ectopic miR-125b expression by using lentivirus reduces myocardial infarct size and preserves cardiac functions in a mouse experimental model of acute myocardial infarction (Wang et al., 2014b). miR-125b is a good cardio-miR that protects the heart from ischemia/reperfusion injury (Figure 3B).

#### Involvement of miR-125b in human cancers

miR-125b is overexpressed in hematological malignancies owing to genetic alterations, such as chromosomal translocation and copy number gain (Table 2). *miR-125b-1* at human chromosome 11q24.1 is upregulated as a result of chromosomal translocation in AML and myelodysplastic syndrome (MDS) with t(2;11)(p21;q24) (Bousquet et al., 2008; Thorsen et al., 2012) and B-cell precursor acute lymphoblastic leukemia (BCP-ALL) with t(11;14)(q24;q32) (Chapiro et al., 2010). miR-125b-2 at human chromosome 21q21.1 is upregulated as a result of copy number gain (21 trisomy) in Down syndrome with acute megakaryocytic leukemia (DS-AMKL) (Klusmann et al., 2010), which leads to the proliferation and self-renewal of hematopoietic progenitors of megakaryocytic and erythroid lineages in part through repression of DICER1 and ST18. miR-125b is also upregulated in childhood ALL with t(12;21)(p13.1;q22) (ETV6/RUNX1-ALL) (Gefen et al., 2010), pancreatic endocrine tumors (Volinia et al., 2006) and urothelial cancer at T2/T3 stages (Veerla et al., 2009). Upregulation of pro-tumor (oncogenic) miR-125b in human cancers promotes proliferation, survival and drug resistance of tumor cells through repression of BAK1, p14, ST18, and TP53 (Figure 3B).

miR-125b is repressed in solid tumors as a result of deletion and epigenetic silencing (Table 2). miR-125b is downregulated in in cervical cancer owing to a deletion of chromosome 11q24.1 that involves *miR-125b-1* (Wilting et al., 2013) and in oral squamous cell carcinoma (OSCC) owing to deletions of chromosome 11q or 21 involving *miR-125b-1* or *miR-125b-2*, respectively (Henson et al., 2009). miR-125b is downregulated in breast cancer (Zhang et al., 2011) and HCC (Alpini et al., 2011) owing to epigenetic silencing induced by CpG hypermetheylation of promoter region(s). miR-125b is also downregulated in prostate cancer (Porkka et al., 2007; Ozen et al., 2008) and colorectal cancer (Chen et al., 2009). Downregulation of anti-tumor (tumor suppressor) miR-125b in human cancers promotes survival, proliferation and invasion of tumor cells through de-repression of BCL2, BCL2L2, E2F3, ERBB2, FGFR2, MCL1, SIRT7, Smoothened and STAT3 (Figure 3B).

miR-125b also functions as an oncogenic or tumor suppressor miRNA in a context-dependent manner (Figure 3B).

### miR-195

#### Human chromosomal locus of miR-195 gene

*miR-195* is derived from the *miR-497/miR-195* locus at human chromosome 17p13.1 (Figure 2).

#### Targets of miR-195

ACVR2A (Bai et al., 2012), ARL2 (ADP-ribosylation factor-like 2) (Zhou et al., 2013c), BCL2 (Liu et al., 2010), BCL2L2 (Yang et al., 2012b), BIRC5 (API4, Apoptosis inhibitor 4) (Itesako et al., 2014), CCND1 (Cyclin D1) (Xu et al., 2009), CCNE1 (Cyclin E1) (Hui et al., 2013), CDC42 (Wang et al., 2013c), CDK4 (Lin et al., 2012b), CDK6 (Xu et al., 2009), E2F3 (Xu et al., 2009), GLUT3 (SLC2A3) (Fei et al., 2012), IKKα (CHUK) (Ding et al., 2013), MYB (Zhou et al., 2014), RAF1 (Li et al., 2011), TAB3 (TAK1/MAP3K7-binding protein 3) (Ding et al., 2013), VAV2 (Wang et al., 2013c), VEGF (Wang et al., 2013c), WEE1 (Bhattacharya et al., 2013), and WNT7A (Itesako et al., 2014) are all validated targets of miR-195 (Table 1).

#### Involvement of miR-195 in cardiovascular diseases

During early post-natal development of mice, miR-195 is upregulated in cardiac ventricles and induces cell-cycle arrest in cardiomyocytes through repression of cell cycle regulators, such as Cdc2a, Chek1, Birc5, Nusap1, and Spag5 (Porrello et al., 2011). Overexpression of miR-195 in the developing heart of transgenic mice by using the β-myosin heavy chain (MHC) promoter gives rise to perinatal cardiomyopathy in one line and ventricular hypoplasia and ventricular septal defects in another line (Porrello et al., 2011). Overexpression of miR-195 in primary neonatal rat cardiomyocytes by using adenoviral vector leads to hypertrophic growth and sarcomeric assembly, and overexpression of miR-195 in the heart of post-natal transgemic mice by using the α-MHC promoter gives rise to cardiac hypertrophy and dilated cardiomyopathy (van Rooij et al., 2006). In transgenic mice with the α-MHC mutation R403Q, miR-195 upregulation and subsequent repression of Cab39 in the heart leads to hypertrophic cardiomyopathy owing to inhibition of Lkb1/Strad/Cab39-dependent AMPK signaling (Chen et al., 2012). Together these facts indicate that miR-195 is a bad cardio-miR that elicits hypertrophic cardiomyopathy, dilated cardiomyopathy and heart failure (Figure 3C).

#### Involvement of miR-195 in cancers

miR-195 is upregulated in metastatic melanoma (Bhattacharya et al., 2013) and some cases of lung cancer (Volinia et al., 2006), colorectal cancer (Ding et al., 2013), prostate cancer (Volinia et al., 2006), gastric cancer (Bandres et al., 2009; Ding et al., 2013) and HCC (Ding et al., 2013). miR-195 can function as an oncogenic miRNA through repression of WEE1 kinase (Figure 3C).

By contrast, miR-195 is preferentially downregulated in breast cancer (Li et al., 2011), gastric cancer (Deng et al., 2013; Ding et al., 2013), colorectal cancer (Chen et al., 2009; Liu et al., 2010; Guo et al., 2013), HCC (Xu et al., 2009; Wang et al., 2013c), bladder cancer (Lin et al., 2012b; Itesako et al., 2014) and prostate cancer (Porkka et al., 2007). *miR-195* is repressed in breast cancer (Li et al., 2011) and gastric cancer (Deng et al., 2013) owing to hypermethylation of CpG islands upstream of the *miR-497/miR-195* locus. *miR-195* is repressed in colorectal cancer owing to deletion of the *miR-497/miR-195* locus (Guo et al., 2013), while *miR-195* is repressed in colorectal adenoma mainly owing to epigenetic silencing and in part owing to deletion (Menigatti et al., 2013). *miR-195* is downregulated in human cancers and pre-cancerous lesions as a result of epigenetic silencing and deletion (Table 2). Because miR-195 is involved in repression of cell cycle accelerators (CCND1, CCNE1, CDK4, CDK6, and E2F3) and anti-apoptotic factors (BCL2, BCL2L2, and BIRC5) (Figure 3C), miR-195 functions as a tumor suppressor miRNA in various types of human cancers.

### miR-214

#### Human chromosomal locus of miR-214 gene

*miR-214* is derived from the *miR-199a-2/miR-214* locus at human chromosome 1q24.3 (Figure 2).

#### Targets of miR-214

ASF1B (Misiewicz-Krzeminska et al., 2013), BCL2L2 (Wang et al., 2013a), ß-catenin (CTNNB1) (Xia et al., 2012), BIM (Zhang et al., 2014), CADM1 (IGSF4A) (Momose et al., 2013), CCL5 (C-C motif ligand 5) (Mitra et al., 2012), CD276 (B7-H3) (Nygren et al., 2014), EZH2 (Derfoul et al., 2011), FGFR1 (Wang et al., 2013b), GALNT7 (N-acetylgalactosaminyltransferase 7) (Peng et al., 2012), HDGF (MGG1L2) (Shih et al., 2012b), ING4 (Zhang et al., 2010), ITGA3 (Integrin α3) (Penna et al., 2011), LTF (Lactoferrin) (Liao et al., 2010), LZTS1 (Xu and Wang, 2014), MAP2K3 (MEK3) (Yang et al., 2009), MAPK8 (JNK1) (Yang et al., 2009), NRAS (Huang et al., 2014a), PSMD10 (Misiewicz-Krzeminska et al., 2013), PTEN (Yang et al., 2008), TFAP2C (AP2γ) (Penna et al., 2011), TP53 (Xu et al., 2012a), TWIST1 (Twist) (Li et al., 2012), UBE2I (UBC9) (Zhao et al., 2012b), and XBP1 (Duan et al., 2012) are all validated targets of miR-214 (Table 1).

#### Involvement of miR-214 in cardiovascular diseases

miR-214 is upregulated as a result of cardiac ischemia and heart failure. In a mouse model of ischemic cardiac injury induced by permanent ligation of the left anterior descending coronary artery, miR-214 prevents cardiomyocyte death owing to Ca^2+^ overload, subsequent cardiac insufficiency and cardiac fibrosis through repression of Slc8a1 (Ncx1, sodium/calcium exchanger), which is the primary Ca^2+^ outflow pump in cardiomyocytes (Aurora et al., 2012). miR-214 protects primary neonatal rat cardiomyocytes from apoptosis induced by ischemia-reperfusion injury and represses Bim, Camk2d (Calmodulin kinase II delta) and Slc8a1 (Aurora et al., 2012). miR-214 also protects primary neonatal rat cardiomyocytes from apoptosis induced by H_2_O_2_ through PTEN repression (Lv et al., 2014). Overexpression of miR-214 in transgenic mice under control of the α-MHC promoter does not induce a deteriorating cardiac phenotype; however, adenovirus-mediated pri-miR-214 delivery and lentivirus-mediated miR-214 delivery induce hypertrophic growth of primary neonatal rat cardiomyocytes in part through Ezh2 repression (van Rooij et al., 2006; Yang et al., 2013). miR-214 is a bi-functional cardio-miR that plays good and bad roles (Figure 3D).

#### Involvement of miR-214 in cancers

Copy number gain of the 1q24.3 region around the *miR-214* locus occurs in 35% of de-differentiated liposarcomas (Tap et al., 2011). miR-214 is upregulated in ovarian cancer (Yang et al., 2008; Xu et al., 2012a), gastric cancer (Volinia et al., 2006; Bandres et al., 2009), pancreatic cancer (Zhang et al., 2010; Jamieson et al., 2012), lung squamous cell carcinoma (Yanaihara et al., 2006), Sézary syndrome (Narducci et al., 2011), liposarcoma (Tap et al., 2011), osteosarcoma (Wang et al., 2014d) and nasopharyngeal cancer (Zhang et al., 2014). miR-214 upregulation in primary gastric cancer occurs as a result of its expression in mesenchymal stem cells (MSCs) rather than cancer cells (Wang et al., 2014a). Genetic alteration as well as tumor-stromal interaction are involved in miR-214 upregulation in human cancers.

Copy number loss of the *miR-214* locus occurs in 24% of breast cancers (Derfoul et al., 2011). miR-214 is downregulated in cervical cancer (Peng et al., 2012; Wang et al., 2013a), HCC (Duan et al., 2012; Shih et al., 2012b), colorectal cancer (Chen et al., 2009), breast cancer (Derfoul et al., 2011), cholangiocarcinoma (Li et al., 2012), glioma (Zhao et al., 2012b), prostate cancer (Srivastava et al., 2013), and bladder cancer (Ratert et al., 2013).

Malignant phenotypes of cancer cells, such as proliferation, survival, drug resistance, invasion and metastasis, are induced by miR-214 upregulation through repression of BIM, CADM1, ING4, PTEN, TFAP2C, and TP53 and also by miR-214 downregulation through de-repression of BCL2L2, ß-catenin, EZH2, FGFR1, GALNT7, HDGF, NRAS, TWIST1, UBE2I, and XBP1 (Figure 3D). miR-214 performs oncogenic functions in some types/subtypes of human cancers and tumor-suppressor functions in other types/subtypes of human cancers.

### Regulatory signaling networks and miRNA-based therapeutics

Regulatory signaling networks are defined as mutual interactions or cross-talks of receptor tyrosine kinase (RTK), G protein-coupled receptor (GPCR) and other receptor signaling cascades (Katoh, 2013a), which are involved in orchestration of fetal development and post-natal homeostasis as well as pathogenesis of non-cancerous and cancerous diseases. WNT, FGF Hedgehog, Notch, TGF-ß, BMP, Nodal, and Activin signaling cascades are major components of the regulatory signaling networks (Bailey et al., 2007; Katoh, 2007; Jayasena et al., 2008; Boulter et al., 2012; Nowell and Radtke, 2013; Coleman et al., 2014).

**Figure 4:**
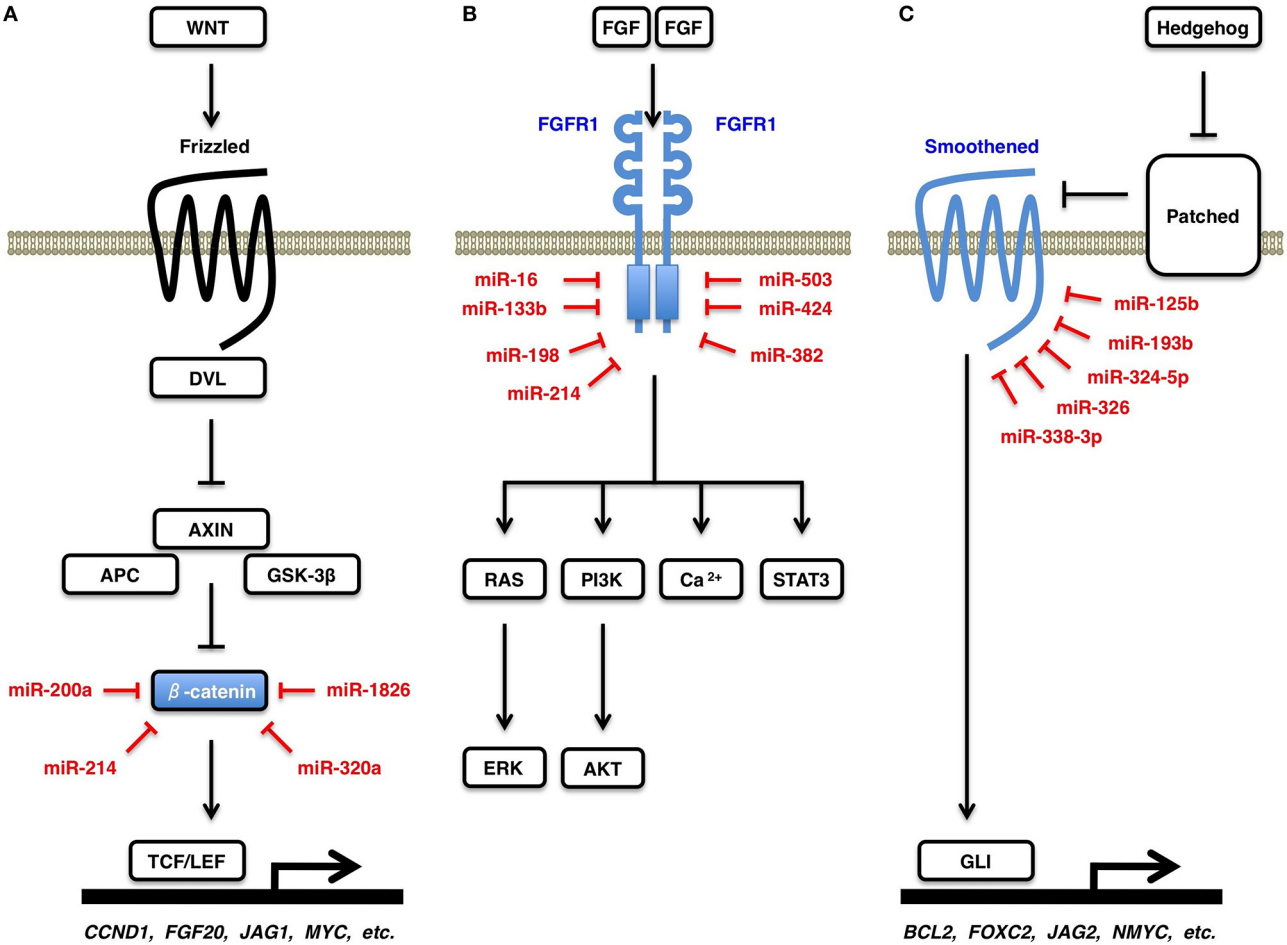
**WNT, FGF, and Hedgehog signaling cascades**. **(A)** Canonical WNT signaling cascade and β-catenin. Canonical WNT signaling activation releases β-catenin from its degradation complex of APC and AXIN, which results in nuclear translocation of β-catenin and transcriptional activation of TCF/LEF-target genes, such as *CCND1 (Cyclin D1), FGF20, JAG1*, and *MYC (c-Myc)*. β-catenin is a direct target of miR-200a, miR-214, miR-320a, and miR-1826. **(B)** FGF signaling cascades and FGFR1. FGF signals induce dimerization and auto-phosphorylation of a receptor tyrosine kinase FGFR1, which activates downstream RAS-ERK, PI3K-AKT, STAT3, and Ca^2+^-release signaling cascades. FGFR1 is a direct target of miR-16, miR-133b, miR-198, miR-214, miR-382, miR-424, and miR-503. **(C)** Hedgehog signaling cascade and Smoothened. Hedgehog signals are transduced from Patched receptor to Smoothened signal transducer, which activates transcription of GLI-target genes, such as *BCL2, FOXC2, JAG2*, and *MYCN* (N-*Myc*). Smoothened is a direct target of miR-125b, miR-193b, miR-324-5p, miR-326, and miR-338-3p.

WNT signals are transduced through Frizzled receptors to the ß-catenin-dependent (canonical) and ß-catenin-independent (non-canonical) cascades (Cohen et al., 2007; Katoh and Katoh, 2007; Klaus and Birchmeier, 2008; Rao and Kühl, 2010). In the absence of canonical WNT signaling, β-catenin is phosphorylated by GSK-3β and is degraded in the proteasome system. By contrast, in the presence of canonical WNT signaling, β-catenin is released from the APC/AXIN degradation complex and activates transcription of canonical WNT target genes, such as *CCND1, FGF20, JAG1*, and *MYC* (Figure 4A). ß-catenin is a direct target of miR-200a (Saydam et al., 2009), miR-214 (Xia et al., 2012), miR-320a (Sun et al., 2012), and miR-1826 (Hirata et al., 2012). Downregulation of miR-200a, miR-214, miR-320a, and miR-1826 de-repress ß-catenin and activate the canonical WNT signaling cascade in human cancers.

FGF signals are transduced to the RAS-ERK, PI3K-AKT, STAT3, and Ca^2+^-release signaling branches through FGFR1 (Figure 4B), FGFR2, FGFR3, and FGFR4 (Turner and Grose, 2010; Goetz and Mohammadi, 2013; Katoh and Nakagama, 2014). FGFR1 is a direct target of miR-16 (Chamorro-Jorganes et al., 2011), miR-133b (Wen et al., 2013), miR-198 (Yang et al., 2014), miR-214 (Wang et al., 2013b), miR-382 (Mor et al., 2013), miR-424 (Chamorro-Jorganes et al., 2011), and miR-503 (Kim et al., 2013b). Upregulation of miR-382 in olfactory neuroepithelium of schizophrenia patients repress FGFR1 (Mor et al., 2013). By contrast, downregulation of miR-133b and miR-214 in human cancers (Wen et al., 2013; Wang et al., 2013b) and that of miR-424 and miR-503 in pulmonary artery epithelial cells of patients with pulmonary arterial hypertension (Kim et al., 2013b) de-repress FGFR1 and promote proliferation of tumor cells and endothelial cells, respectively, through FGF signaling activation.

Hedgehog signals are transduced from Patched receptors to Smoothened signal transducer, which activates GLI-dependent transcription of target genes, such as *BCL2, FOXC2, JAG2*, and *MYCN (*N*-Myc)* (Figure 4C). Hedgehog-Smoothened-GLI signaling cascade is involved in the regulation of cellular survival, proliferation, motility and stemness (Jiang and Hui, 2008; Katoh and Katoh, 2009; Lin and Matsui, 2012a). Smoothened is a direct target of miR-125b (Ferretti et al., 2008), miR-193b (González-Gugel et al., 2013), miR-324-5p (Ferretti et al., 2008), miR-326 (Ferretti et al., 2008), and miR-338-3p (Huang et al., 2011b). Downregulation of miR-125b, miR-193b, miR-324-5p, miR-326, and miR-338-3p in human cancers de-repress Smoothened and promotes tumor proliferation and invasion through aberrant Hedgehog signaling activation.

miRNAs are therapeutic targets for non-cancerous diseases as well as cancers, because disease-related miRNAs dysregulate the regulatory signaling networks (Katoh and Katoh, 2008; Mo et al., 2013; Parpart and Wang, 2013; Katoh et al., 2013c). Reduction of elevated pro-disease miRNA and restoration of declined anti-disease miRNA are two major strategies of miRNA-based therapeutics. Locked-nucleic-acid-modified anti-miRNA oligonucleotides (LNA-antimiRs) are utilized for the reduction of pro-disease miRNAs, while adenovirus and letivirus vectors are utilized for the restoration of anti-disease miRNAs (Ji et al., 2008; Kasinski and Slack, 2011; Shi et al., 2011; van Rooij and Olson, 2012). Reduction of FGFR1-targeting miRNAs for cancer therapy deteriorate diabetes and cardiac functions, because the FGFR1-PI3K-AKT signaling cascade is involved in cancer promotion (Katoh et al., 2013c) as well as diabetes control (Suh et al., 2014). By contrast, restoration of miRNA targeting BAK1, BIM, or PTEN for cardiomyocyte protection promotes survival of tumor cells (Figure 3). miRNA-based therapy is at the risk of adverse effects owing to repression of verified targets in different disciplines. In addition, because multiple miRNAs repress the same target (Figure 4) and each miRNA represses multiple targets (Table 1), miRNA-based therapy is also at the risk of adverse effects owing to repression of unidentified targets in individual patients. There are many obstacles before clinical application of miRNA-based therapeutics.

### Circulating miR-24, miR-125b, miR-195, and miR-214

miRNAs function within the cell where they were produced as well as in other cells that receive miRNAs secreted or released from the cell of their origin (Valadi et al., 2007; Skog et al., 2008). Extracellular miRNAs are detected in various types of body fluids, such as blood, tears, saliva, urine, vitreous humor, cerebro-spinal fluid, pleural fluid, peritoneal fluid, seminal fluid, breast milk, and amniotic fluid (Mitchell et al., 2008; Weber et al., 2010; Ragusa et al., 2013). Extracellular miRNAs are classified into miRNAs in the blood (circulating miRNAs) and those in other body fluids. Because circulating miRNAs within exosomes (Taylor and Gercel-Taylor, 2008), microvesicles (Hunter et al., 2008) and high-density lipoprotein (Vickers et al., 2011) or those conjugated with AGO2 protein (Arroyo et al., 2011) are stable, circulating miRNAs are going to be utilized as diagnostics and prognostic biomarkers (Table 3).

**Table 3:** **Circulating miR-24, miR-125b, miR-195, and miR-214 in diseases**.

**Circulating miRNA**	**Disease**
miR-24 Up	Breast cancer
	Lung cancer
	Malignant peripheral nerve sheath tumor with NF1 mutation
	Multiple system atrophy
	Osteoporotic fracture
	Parkinson's disease
	Preeclamptic pregnancy
	Rheumatoid arthritis
	Type 1 diabetes
	Type 2 diabetes
miR-24 Down	Type 2 diabetes
miR-125b Up	Breast cancer
	Non-alcoholic fatty liver disease
	Non-small-cell lung cancer
	Osteoporotic fracture
	Rheumatoid arthritis
miR-125b Down	Acute myocardial infarction
	Alzheimer's disease
	Atopic dermatitis
	Chronic kidney disease
	Melanoma
	Morbidly obese
	Psoriasis vulgaris
	Type 2 Diabetes
miR-195 Up	Acute myocardial infarction
	Breast cancer
	Colorectal adenoma
	Prostate cancer
miR-195 Down	Adrenocortical carcinoma
	Hepatocellular carcinoma
	Schizophrenia
	Type 2 Diabetes
miR-214 Up	Breast cancer
	Malignant peripheral nerve sheath tumor
	Ovarian cancer
miR-214 Down	Acute myocardial infarction
	Angina pectoris

Circulating miR-24 is elevated in patients with breast cancer (Wu et al., 2012b; Sochor et al., 2014), lung cancer (Le et al., 2012), malignant peripheral nerve sheath tumor with the NF1 mutation (Weng et al., 2013), multiple system atrophy (Vallelunga et al., 2014), osteoporotic fracture (Seeliger et al., 2014), Parkinson's disease (Vallelunga et al., 2014), preeclamptic pregnancy (Wu et al., 2012a), rheumatoid arthritis (Murata et al., 2013) and type 1 diabetes (Nielsen et al., 2012). Wang et al. reported elevated miR-24 in type 2 diabetes patients (Wang et al., 2014c), whereas Zampetaki et al. reported reduced miR-24 in type 2 diabetes patients (Zampetaki et al., 2010).

Circulating miR-125b is elevated in patients with breast cancer (Wang et al., 2012a; Mar-Aguilar et al., 2013), non-alcoholic fatty liver disease (Pirola et al., 2014), non-small-cell lung cancer (Yuxia et al., 2012; Cui et al., 2013), osteoporotic fracture (Seeliger et al., 2014) and rheumatoid arthritis (Duroux-Richard et al., 2014), whereas circulating miR-125b is reduced in patients with acute myocardial infarction (Huang et al., 2014b), Alzheimer's disease (Tan et al., 2014), atopic dermatitis (Koga et al., 2014), chronic kidney disease (Chen et al., 2013b), melanoma (Alegre et al., 2014), morbidly obese (Ortega et al., 2013), psoriasis vulgaris (Koga et al., 2014), and type 2 diabetes (Ortega et al., 2014).

Circulating miR-195 is elevated in patients with acute myocardial infarction (Long et al., 2012), breast cancer (Heneghan et al., 2010), colorectal adenoma (Kanaan et al., 2013), and prostate cancer (Mahn et al., 2011), whereas circulating miR-195 is reduced in adrenocortical carcinoma (Chabre et al., 2013), HCC (Qu et al., 2011), schizophrenia (Shi et al., 2012), and type 2 diabetes (Ortega et al., 2014).

Circulating miR-214 is elevated in patients with breast cancer (Schwarzenbach et al., 2012), malignant peripheral nerve sheath tumor (Weng et al., 2013) and ovarian cancer (Taylor and Gercel-Taylor, 2008), whereas circulating miR-214 is reduced in patients with acute myocardial infarction and angina pectoris (Lu et al., 2013).

These facts clearly indicate that circulating miRNAs reported as cancer biomarkers are also dysregulated in non-cancerous diseases, and that miRNAs reported as biomarkers of non-cancerous diseases are also dysregulated in cancers (Table 3).

### miRNA regulation by genetic and environmental factors

**Figure 5:**
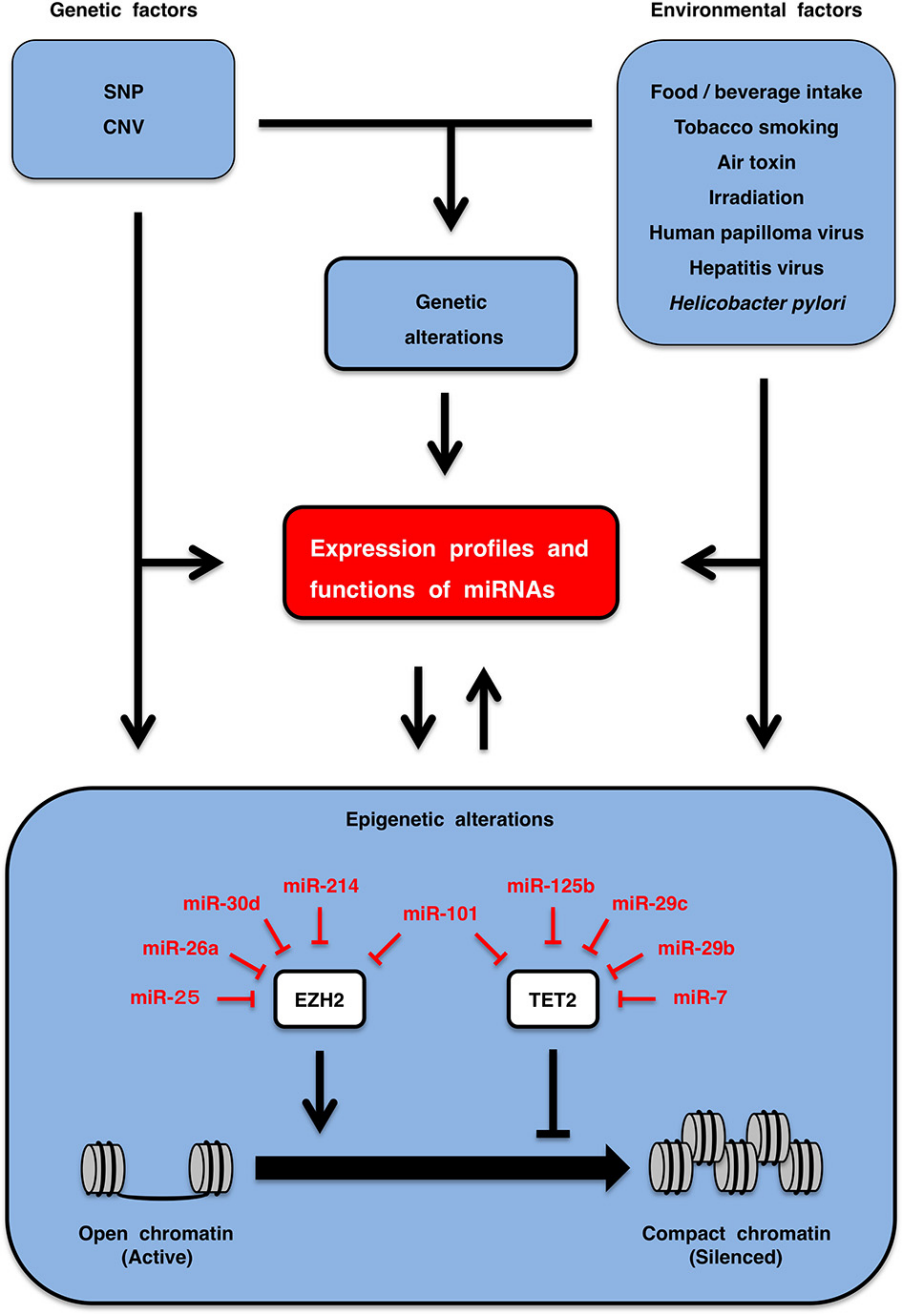
**Regulation of circulating miRNAs**. Genetic factors, such as single nucleotide polymorphism (SNP) and copy number variation (CNV), as well as environmental factors, including life style (food/beverage intake, tobacco smoking, air toxin, irradiation, *etc*.) and chronic infection (human papilloma virus, hepatitis virus, *Helicobacter pylori, etc*.) are involved in the regulation of expression profiles and functions of miRNAs (upper part). EZH2 and TET2 are epigenetic regulators that are involved in inactivation and activation of genes through repressive histone marking and CpG-island de-methylation, respectively. miRNA expression is downregulated by epigenetic silencing, while epigenetic regulators are repressed by multiple miRNAs. miRNAs and epigenetics are in the relationship of mutual regulation (lower part). Genetic and environmental factors regulate circulation miRNA profiles directly as well as indirectly through genetic and epigenetic alterations.

Genetic factors are associated with individual traits and disease susceptibility (Lichtenstein et al., 2000; Zimmet et al., 2001; Milne et al., 2009). Single nucleotide polymorphisms (SNPs) and copy number variations (CNVs) are major germ-line variations. The SNP rs1434536 is located in the miR-125b-binding site within the 3′-untranslated region (UTR) of BMPR1B. The C and T alleles of the rs1434536 SNP are sensitive and resistant to BMPR1B repression by miR-125b, respectively (Sætrom et al., 2009). The homozygous T genotype of rs1434536 is associated with increased risk of breast cancer (Sætrom et al., 2009) and decreased risk of endometriosis (Chang et al., 2013). Copy number loss of the miR-195 locus occurs in autism patients (Vaishnavi et al., 2013). Copy number gain of the miR-125b-2 locus occurs in Down syndrome patients as a result of trisomy 21, which leads to elevated circulating miR-125b in pregnant women with a Down syndrome fetus (Kotlabova et al., 2013) and causes acute megakaryocytic leukemia in Down syndrome patients (Klusmann et al., 2010). Genetic factors directly affect expression profiles and functions of miRNAs (Figure 5, upper left).

Environmental factors are also associated with disease susceptibility (Lichtenstein et al., 2000; Zimmet et al., 2001). Life style (food/beverage intake, tobacco smoking, air toxin, irradiation, *etc*.) and chronic infection (papilloma virus, hepatitis virus, *Helicobater pylori, etc*.) are environmental factors affecting individuals. Human miR-125b is downregulated in the bronchial epithelium of current smokers compared with never smokers (Schembri et al., 2009), and rat miR-125b is downregulated in the lungs of rats that were exposed to environmental smoke for 28 days (Izzotti et al., 2009). The expression profile of miRNAs in airway epithelial cells is altered by air toxins, such as diesel exhaust particles and formaldehyde (Jardim et al., 2009; Rager et al., 2011), whereas that in breast cancer cells is altered by endocrine disruptors, such as o,p'-dichlorodiphenyltrichloroethane (DDT), bisphenol A (BPA), fenhexamid and fludioxonil (Tilghman et al., 2012; Teng et al., 2013). The circulating miRNA landscape is altered by total-body γ-irradiation (Jacob et al., 2013) and by uptake of dietary polyphenols, such as quercetin, hesperidin, naringenin, anthocyanin, catechin, proanthocyanin, caffeic acid, ferulic acid and curcumin (Milenkovic et al., 2012), in model animal experiments. miRNA expression profile is altered by chronic infection with human papilloma virus, hepatitis virus and *Helicobacter pylori*, which are involved in pathogenesis of cervical cancer (Wang et al., 2008b), HCC (Ladeiro et al., 2008; Arzumanyan et al., 2013), and gastric cancer (Zhang et al., 2008), respectively. Environmental factors directly alter circulating or tissue levels of miRNAs (Figure 5, upper right).

Genetic alterations, such as gene amplification, deletion, translocation, point mutation or single nucleotide variation (SNV), occur in tumor cells during multi-stage carcinogenesis owing to mutual interactions of genetic and environmental factors (Lichtenstein et al., 2000; Katoh et al., 2013c; Katoh and Nakagama, 2014). SNVs in diffuse large B-cell lymphomas that disrupt the miR-125b-binding site within the 3′-UTR of TP53 are associated with better prognosis of patients owing to de-repression of a tumor suppressor TP53 (Li et al., 2013b). Effects of gene amplification, deletion and translocation on expression profiles of miR-24, miR-125b, miR-195, and miR-214 have been described above (Table 2). Genetic alterations play a key role for the regulation of miRNA profiles in somatic cells (Figure 5, upper middle).

Epigenetics is chromatin-based genomic regulations that are involved in the modulation of expression landscapes of mRNAs and miRNAs during fetal development, post-natal homeostasis and pathogenesis of human diseases (Datta et al., 2008; Kulis and Esteller, 2010; Ordovás and Smith, 2010; Baylin and Jones, 2011; Dawson and Kouzarides, 2012). EZH2 and TET2 are representative epigenetic regulators that are repressed by miR-214 and miR-125b, respectively (Table 1). EZH2 is a human homolog of *Drosophiula* Enhancer of zeste, which is a component of the Polycomb repressive complex 2 (PRC2) and PRC2-like complex (Sparmann and van Lohuizen, 2006). EZH2 is involved in epigenetic silencing of PRC target genes through trimethylation of histone H3 lysine 27 (H3K27me3) and CpG hypermethylation of promoters (Figure 5, lower part). Because EZH2 is a target of miR-25 (Esposito et al., 2012), miR-26a (Sander et al., 2008), miR-30d (Esposito et al., 2012), miR-101 (Varambally et al., 2008), and miR-214 (Derfoul et al., 2011), downregulation of miR-25, miR-26a, miR-30d, miR-101, and miR-214 in human cancers are associated with EZH2 upregulation and malignant phenotypes. TET2 is involved in promoter de-methylation through enzymatic conversion of 5-methylcytosine (5mC) to 5-hydroxylmethyl-cytosine (5hmC) (Ito et al., 2010). Loss-of-function *TET2* mutations occur in patients with myeloproliferative neoplasms, MDS and AML (Shih et al., 2012a), while upregulation of TET2-targeting miRNAs, such as miR-7, miR-29b, miR-29c, miR-101, and miR-125b, occur in AML patients with wild-type *TET2* (Cheng et al., 2013). miRNAs targeting EZH2 and TET2 alter epigenetic regulations of disease-associated genes. By contrast, disease-associated miRNAs are epigenetically silenced owing to promoter CpG hypermethylation in human diseases (Table 2). Epigenetic alterations also play a key role for the regulation of miRNA profiles in somatic cells (Figure 5, lower part).

Genetic and environmental factors dynamically alter expression profiles of miRNAs in individuals and also indirectly alter miRNA profiles through genetic and epigenetic alterations in patients with non-cancerous diseases and cancers (Figure 5).

### Circulating miRNA-based diagnostics

Circulating miR-195 is upregulated in colorectal adenoma (Kanaan et al., 2013); however, miR-195 in colorectal adenoma tissues is repressed owing to epigenetic silencing and deletion (Menigatti et al., 2013). Circulating miR-125b, miR-195, and miR-214 are upregulated in breast cancer patients (Table 2), whereas these miRNAs in breast cancer tissues are downregulated owing to epigenetic silencing or deletion (Table 2). These facts clearly indicate that circulating miRNAs in cancer patients are not always derived from tumor tissues.

**Figure 6:**
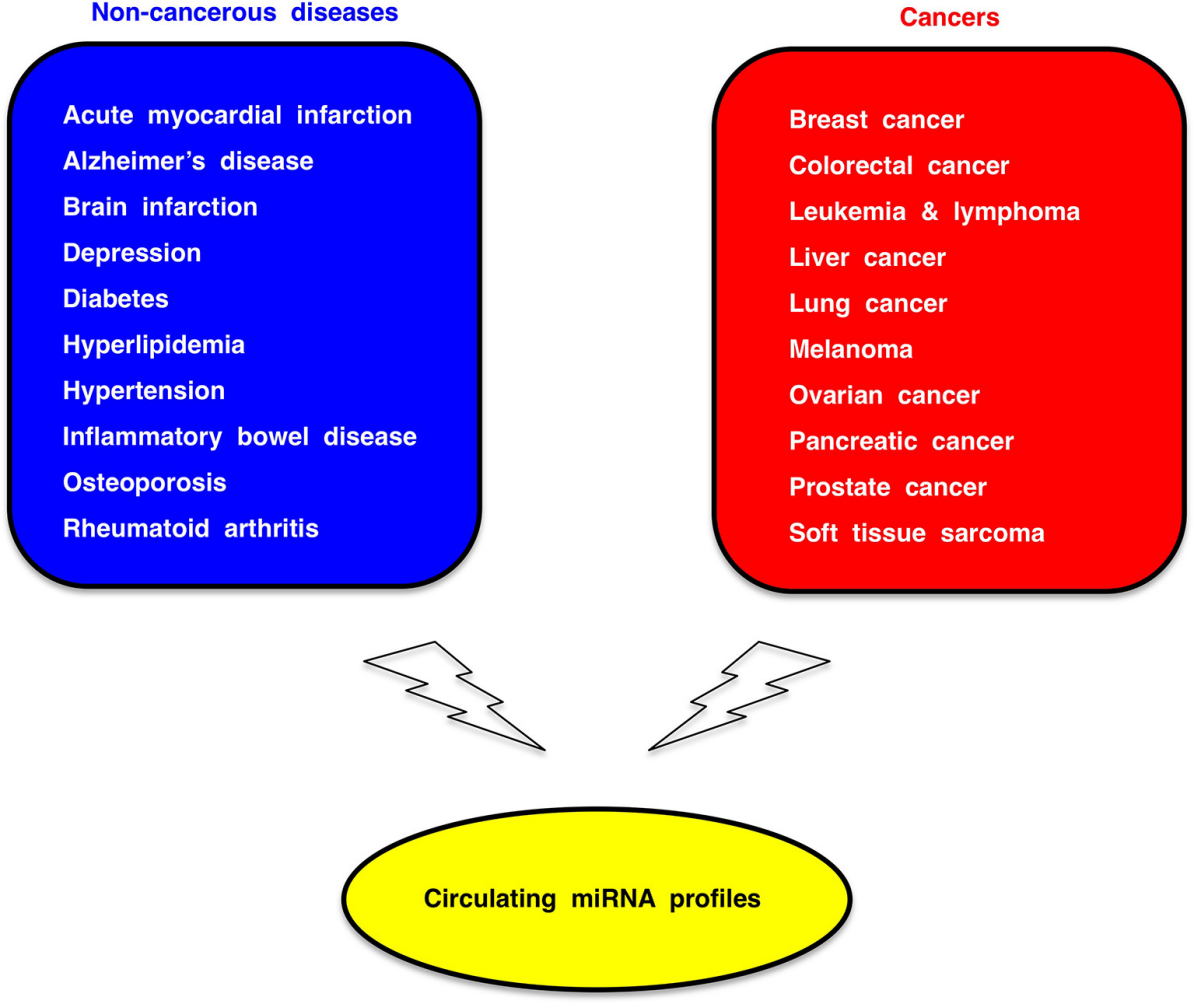
**Circulating miRNA association study (CMAS)**. Dysregulation of circulating miRNAs occur in a variety of human disease. Circulating miRNA profiles in several thousands of cases each for non-cancerous common diseases (blue box) and major cancers (red box) should be investigated for the establishment of miRNA-based diagnostic platform.

Because circulating miRNA profiles are dynamically regulated by genetic and environmental factors (Figure 5), circulating miRNA profiles of cancer patients reflect co-existing non-cancerous diseases or individual whole-body conditions. Therefore, circulating miRNA association studies (CMASs) within several thousands of cases each for common non-cancerous diseases as well as major cancers (Figure 6) should be carried out to establish a reliable and robust platform of miRNA-based diagnostics.

## Conclusion

Cardio-miRs and onco-miRs bear some similarities in functions and circulation profiles. miRNAs modulate the regulatory signaling networks that are involved in orchestration of embryogenesis and homeostasis as well as pathogenesis of human diseases. Circulating miRNA profiles within several thousands of cases each for non-cancerous and cancerous diseases are necessary for the establishment of miRNA-based diagnostics.

### Conflict of interest statement

The author declares that the research was conducted in the absence of any commercial or financial relationships that could be construed as a potential conflict of interest.
